# Ecto-GPR37: a potential biomarker for Parkinson’s disease

**DOI:** 10.1186/s40035-021-00232-7

**Published:** 2021-02-26

**Authors:** Xavier Morató, Paula Garcia-Esparcia, Josep Argerich, Franc Llorens, Inga Zerr, Wojciech Paslawski, Eva Borràs, Eduard Sabidó, Ulla E. Petäjä-Repo, Víctor Fernández-Dueñas, Isidro Ferrer, Per Svenningsson, Francisco Ciruela

**Affiliations:** 1 Pharmacology Unit, Department of Pathology and Experimental Therapeutics, Faculty of Medicine and Health Sciences, Institute of Neurosciences, University of Barcelona, L’Hospitalet de Llobregat, Spain; 2 Neuroscience Program, Bellvitge Biomedical Research Institute, IDIBELL, L’Hospitalet de Llobregat, Spain; 3 Section of Neurology, Department of Clinical Neuroscience, Department of Neuroscience, Karolinska Institute, Stockholm, Sweden; 4 Neuropathology Unit, Department of Pathology and Experimental Therapeutics, Faculty of Medicine and Health Sciences, Institute of Neurosciences, University of Barcelona, L’Hospitalet de Llobregat, Spain; 5 CIBERNED, Centro de Investigación Biomédica en Red de Enfermedades Neurodegenerativas, Instituto Carlos III, Madrid, Spain; 6 Department of Neurology, Clinical Dementia Center and National Reference Center for CJD Surveillance, University Medical School, Göttingen, Germany; 7 German Center for Neurodegenerative Diseases, Göttingen, Germany; 8 Proteomics Unit, Center for Genomic Regulation, Barcelona, Spain; 9 Proteomics Unit, Universitat Pompeu Fabra, Barcelona, Spain; 10 Research Unit of Biomedicine, Medical Research Center Oulu, University of Oulu, Oulu, Finland

**Keywords:** GPR37, Pael-R, Parkinson’s disease, Cerebrospinal fluid, Biomarker, Alzheimer’s disease, α-Synuclein, Orphan receptor

## Abstract

**Objective:**

α-Synuclein has been studied as a potential biomarker for Parkinson’s disease (PD) with no concluding results. Accordingly, there is an urgent need to find out reliable specific biomarkers for PD. GPR37 is an orphan G protein-coupled receptor that toxically accumulates in autosomal recessive juvenile parkinsonism. Here, we investigated whether GPR37 is upregulated in sporadic PD, and thus a suitable potential biomarker for PD.

**Methods:**

GPR37 protein density and mRNA expression in postmortem substantia nigra (SN) from PD patients were analysed by immunoblot and RT-qPCR, respectively. The presence of peptides from the N-terminus-cleaved domain of GPR37 (i.e. ecto-GPR37) in human cerebrospinal fluid (CSF) was determined by liquid chromatography-mass spectrometric analysis. An engineered in-house nanoluciferase-based immunoassay was used to quantify ecto-GPR37 in CSF samples from neurological control (NC) subjects, PD patients and Alzheimer’s disease (AD) patients.

**Results:**

GPR37 protein density and mRNA expression were significantly augmented in sporadic PD. Increased amounts of ecto-GPR37 peptides in the CSF samples from PD patients were identified by mass spectrometry and quantified by the in-house ELISA method. However, the CSF total α-synuclein level in PD patients did not differ from that in NC subjects. Similarly, the cortical GPR37 mRNA expression and CSF ecto-GPR37 levels in AD patients were also unaltered.

**Conclusion:**

GPR37 expression is increased in SN of sporadic PD patients. The ecto-GPR37 peptides are significantly increased in the CSF of PD patients, but not in AD patients. These results open perspectives and encourage further clinical studies to confirm the validity and utility of ecto-GPR37 as a potential PD biomarker.

**Supplementary Information:**

The online version contains supplementary material available at 10.1186/s40035-021-00232-7.

## Background

Parkinson’s disease (PD) is the second most common type of neurodegenerative disorder [1], with hallmarks of Lewy body (LB) formation and neuronal death and synaptic loss in the substantia nigra (SN) [2]. Cerebrospinal fluid (CSF) is an accessible source of brain-derived proteins and metabolites, which mirrors molecular changes that occur in the central nervous system. The decreased CSF levels of amyloid-β 42 (Aβ42) and altered content of tau protein species are used in clinical practice to aid diagnosis of Alzheimer’s disease (AD) [3]. In contrast, although potential diagnostic/prognostic CSF biomarkers of PD (i.e. α-synuclein species, lysosomal enzymes, markers of Aβ and tau pathology, and neurofilament light chain) have been largely assessed, no conclusive data have been obtained. However, the combined assessment of some of these biomarkers in PD patients can provide more accurate diagnostic and prognostic results, which necessitate systematic studies confirming the validity and utility of combinations of certain CSF (and blood) PD/AD biomarkers for clinical practice [4, 5].

GPR37 is an orphan G protein-coupled receptor (GPCR) expressed in brain regions such as cerebellum, corpus callosum, caudate nucleus, putamen, hippocampus and SN [6, 7]. The physiological function of this receptor has yet to be elucidated. A recent study has shown that GPR37 mediates the control of oligodendrocyte differentiation [8], but there is little information on the role of GPR37 in other glial cells and neurons. GPR37 is also known as a parkin-associated endothelin-receptor-like receptor (Pael receptor) [9] as it was originally identified as a substrate of parkin, an E3 ubiquitin ligase encoded by the *PARK2* gene involved in ubiquitination and proteasome-mediated degradation/clearance of misfolded proteins [9–11]. Mutations in the *PARK2* gene that lead to the loss of the ubiquitin ligase activity of parkin are the most common cause of autosomal recessive juvenile parkinsonism [10]. Consistently, an insoluble form of GPR37 has been reported to accumulate in the brains of AR-JP patients [9], suggesting a neuropathological role of this receptor in PD. In line with this, mice lacking GPR37 are partially protected against dopaminergic cell death caused by 1-methyl-4-phenyl-1,2,3,6-tetrahydropyridine [12]. Nevertheless, in heterologous expression systems, GPR37 matures and is exported from the endoplasmic reticulum to the cell surface, where its long extracellular N-terminus is rapidly and constitutively cleaved by metalloproteinases (MPs) [13]. Thus, the GPR37 ectodomain (i.e. ecto-GPR37) is released from cells by shedding, a phenomenon rarely described for GPCRs. Importantly, MPs have received much attention since they can cleave a large number of protein substrates (i.e. α-synuclein, amyloid precursor protein, and Apolipoprotein E receptor), thus playing a potential role in neurodegenerative diseases. Indeed, altered levels of MPs have been described in post-mortem brain samples from PD [14] and AD [15, 16] subjects.

Here, we set out to assess the expression of GPR37 in sporadic PD and the presence of ecto-GPR37 peptides in CSF of PD patients. We engineered an in-house immunoassay to assess ecto-GPR37 levels in CSF samples from two different cohorts of PD patients along with age- and gender-matched neurological control (NC) subjects. The CSF ecto-GPR37 levels in AD subjects were also tested.

## Methods

### Antibodies

The following antibodies were used: rabbit anti-human-GPR37-N polyclonal antibody [17], rabbit anti-mouse-GPR37-N polyclonal antibody [7, 18], rabbit anti-pan-GPR37-C polyclonal antibody [18], mouse anti-β-actin monoclonal antibody (A5316; Sigma-Aldrich, St. Louis, MO) and rabbit anti-α-actinin polyclonal antibody (Santa Cruz Biotechnology Inc., Dallas, TX).

### Human post-mortem brain samples

Human post-mortem brain tissues and CSF samples were obtained from the Institute of Neuropathology HUB-ICO-IDIBELL Biobank and the Hospital Clinic-IDIBAPS Biobank following the guidelines of Spanish legislation on this matter (Real Decreto de Biobancos 1716/2011) [19, 20] and upon approval of the local ethics committees. The pathological cases were categorized as PD (LB disease pathology) or AD (neurofibrillary tangle [NFT] pathology) at stages 1–6 according to the nomenclature of Braak et al. [21, 22]. The post-mortem delay (PMD) between death and tissue processing was between 2.5 h and 20.35 h. One hemisphere was immediately cut into 1-cm-thick coronal sections, and selected areas of the encephalon were rapidly dissected, frozen on metal plates over dry ice, placed in individual air-tight plastic bags and stored at − 80 °C until use for biochemical studies. The other hemisphere was fixed by immersion in 4% buffered formalin for 3 weeks for morphological studies. The neuropathological diagnosis of all cases was made based on the evaluation of 20 selected de-waxed paraffin sections containing regions of the cerebral cortex, diencephalon, thalamus, brain stem and cerebellum, which were stained with haematoxylin, eosin and Klüver-Barrera. Only cases categorized as stages 3, 4, 5 and 6 (i.e. PD or AD stage 3–6) were considered. Cases with concomitant tauopathies with NFT and A-B stages of Aβ plaque [22] were excluded from the present study. The middle-aged control cases had not suffered from neurologic, psychiatric, or metabolic diseases (including metabolic syndrome), and did not have abnormalities in the neuropathological examination excepting stages 1–2 of NFT pathology and phases A-B of Aβ plaques. Cases with associated pathologies such as vascular disease (except mild atherosclerosis and arteriolosclerosis), TDP-43 proteinopathy, infections of the nervous system, brain neoplasm, systemic and central immune diseases, metabolic syndrome and hypoxia were excluded from the present study. The selected brain areas were SN, frontal cortex (FC) and entorhinal cortex (EC), and different series of samples (i.e. NCs, Braak PD/AD stage 3, Braak PD/AD 4, Braak PD/AD 5 and Braak PD/AD 6) were prepared according to their experimental use: (i) PD SN immunoblotting: 5 NCs (3 males and 2 females; mean age: 84.2 ± 7 years; PMD: 10.7 ± 7.7 h), 5 Braak PD stage 4 (3 males and 2 females; mean age: 77.6 ± 7.6 years; PMD: 10.1 ± 8.2 h), 5 Braak PD stage 5 (3 males and 2 females; mean age: 80.6 ± 6.4 years; PMD: 10.8 ± 5.2 h) and 5 Braak PD stage 6 (3 males and 2 females; mean age: 76.2 ± 7.25 years; PMD: 9.8 ± 5.5 h) cases; (ii) PD SN RT-qPCR: 10 NCs (8 males and 2 females; mean age: 61.4 ± 12.8 years; PMD: 5.7 ± 3.5 h; RNA integrity number [RIN]: 6.6 ± 0.4), 7 Braak PD stage 3/4 (3 males and 4 females; mean age: 78 ± 5.9 years; PMD: 5.5 ± 1.2 h; RIN: 7.2 ± 0.5) and 17 Braak PD stage 5/6 (12 males and 5 females; mean age: 79.8 ± 4.9 years; PMD: 8.6 ± 3.4 h; RIN: 7.1 ± 0.6) cases; (iii) AD FC RT-qPCR: 17 NCs (10 males and 7 females; mean age: 61.5 ± 14.4 years; PMD: 5.1 ± 1.9 h; RIN: 7.5 ± 0.6), 9 Braak AD stage 3/4 (4 males and 5 females; mean age: 78.6 ± 8.1 years; PMD: 6.7 ± 3.5 h; RIN: 7.7 ± 1) and 20 Braak AD stage 5/6 (9 males and 11 females; mean age: 80.5 ± 6.9 years; PMD: 9.3 ± 5.8 h; RIN: 7.5 ± 0.8); and (iv) AD EC RT-qPCR: 5 NCs (4 males and 1 female; mean age: 41.7 ± 9.7 years; PMD: 5.5 ± 0.8 h; RIN: 6.3 ± 0.9), 4 Braak AD stage 3/4 (3 males and 1 female; mean age: 80.5 ± 1 years; PMD: 6.6 ± 4.2 h; RIN: 6.2 ± 0.7) and 5 Braak AD stage 5/6 (3 males and 2 females; mean age: 79.2 ± 5.1 years; PMD: 7.9 ± 4 h; RIN: 6.2 ± 0.8).

### Participants

PD patients were recruited at the movement disorders clinic at Karolinska University Clinic, Huddinge, Sweden. Patients underwent a clinical examination (including ratings of motor, cognitive and psychiatric symptoms) by an experienced neurologist (PS) (Table 1). All the included patients were followed longitudinally and PD diagnosis, including subsequent responsiveness to dopaminergic therapy, was confirmed before they were included in the CSF analysis. Hence, the PD patients satisfied the clinical diagnosis of PD according to the U.K. Parkinson Disease Society Brain Bank diagnostic criteria. Motor symptoms were evaluated with the Unified Parkinson’s Disease Scale (UPDRS) part 3 in the ON state of medication cycle. Non-motor and motor symptoms in daily activities were evaluated with UPDRS part 1 and part 2, respectively. UPDRS part 4 was used to measure motor complications [23]. Almost all patients had available scores for Hoehn & Yahr scale (*n* = 40) assessing motor impairment [24], and 66% of the patients had available Montreal Cognitive Assessment scores (MoCA, *n* = 28) evaluating the cognitive impairment [25]. In addition, within the routine visit self-evaluation questionnaires were implemented, including Non-Motor Symptoms Questionnaire (*n* = 26) [26], and Hospital Anxiety and Depression scale (*n* = 26) [27]. Finally, medications were presented as *L*-dopa equivalent doses [28]. Thirteen patients were drug-naïve, de novo, patients at the time of lumbar puncture. Age- and gender-matched control subjects were recruited from routine neurology examinations. Control patients were also recruited at Karolinska University Clinic, Huddinge, Sweden and were diagnosed with benign neurological diagnoses, such as tension headache with no evidence of dementia, no cognitive complaints, no PD or other brain disease, and thus were defined as NCs.

**Table 1:** Demographic and clinical characteristics of the study participants. Participants were recruited at the movement disorders clinic at Karolinska University Clinic, Huddinge, Sweden. Patients underwent a clinical examination as described in the Methods section

	NC	PD
Age (years)	64 ± 12	64 ± 11
Sex (M/F)	27/18	23/18
Disease duration (years)	—	5.8 ± 5.5
UPDRS Total (*n* = 26)	—	42 ± 18
UPDRS III (*n* = 26)	—	26 ± 14
H&Y score (*n* = 40)	—	II
MoCA (*n* = 28)	—	23 ± 5
HADS (*n* = 26)	—	14 ± 8
LED (*n* = 28)	—	709 ± 512

Data are expressed as mean **±** SD except the Hoehn and Yahr (H&Y) score as median. *UPDRS* Unified Parkinson’s Disease Scale; *MoCA* Montreal Cognitive Assessment scores; *HADS* Hospital Anxiety and Depression scale; *LED L*-dopa equivalent dose

### CSF samples

For preliminary assessment of ecto-GPR37 in humans, we used post-mortem CSF samples from eight PD pathological cases (six males and two females, mean age: 71.4 ± 9.9 years; post-mortem interval: 6.6 ± 3.7 h) (neuropathology confirmed from Bellvitge Hospital Neuropathology Service, see above) and eight age-matched control cases (all male; mean age: 66.8 ± 5.2 years; post-mortem interval: 6.4 ± 2.3 h). Subsequently, CSF samples were obtained by lumbar puncture from participants from Karolinska University Clinic, collected into polypropylene-tubes and immediately centrifuged at 1800 × g at 4 °C for 10 min before being stored in aliquots of 100 μl at − 80 °C as previously described [29]. Thus, CSF samples from a total of 86 participants (45 NCs and 41 PD patients out of whom 12 were drug-naïve) were used in the study.

For the analysis of CSF ecto-GPR37 in AD, NC (9 males and 13 females; mean age: 65.7 ± 6.7 years) and AD (10 males and 13 females; mean age: 67.6 ± 7.9 years) participants were enrolled at the Department of Neurology, Clinical Dementia Center of Göttingen (Germany). The NC participants were those diagnosed with non-primarily neurodegenerative neurological and psychiatric diseases. AD was diagnosed according to the guideline of the National Institute on Aging–Alzheimer’s Association workgroups [30]. All the participants had given consent for the use of their clinical data and CSF for research purposes, and the study was approved by the regional ethics committee for medical research in Stockholm (2011/500–31/1 and 2012/2224–32/4). All procedures followed were in accordance with the ethical standards indicated by the Declaration of Helsinki of 1975, as revised in 2000.

### Animals

C57BL/6 J wild-type (GPR37^+/+^) and GPR37-KO (GPR37^−/−^) male mice (Strain Name: B6.129P2-*GPR37*^*tm1Dgen*^/J; The Jackson Laboratory, Bar Harbor, ME) at 8 weeks of age were used [7]. Animal protocols were approved by the University of Barcelona Committee on Animal Use and Care. Animals were housed and tested in compliance with the guidelines described in the Guide for the Care and Use of Laboratory Animals [31] and following the European Union directives (2010/63/EU), Federation for Laboratory Animal Science Associations, and Animal Research: Reporting In Vivo Experiments guidelines. All efforts were made to minimize animal suffering and the number of animals used. All animals were housed in groups of five in standard cages with ad-libitum access to food and water under a 12-h dark/light cycle (starting at 7:30 AM) at 22 °C with 66% humidity (standard conditions).

### Mouse CSF samples

Mice were anesthetized by intraperitoneal administration of a mixture of ketamine (100 mg/kg, Merial, Spain)/xylazine (10 mg/kg, Calier, Spain). The animal was stereotaxically immobilized to facilitate the access to the cisterna magna through a sagittal incision just behind the occiput. CSF (~ 10 μl) was collected by puncturing the cisterna magna with a micro-fine (30G) 3-mL syringe-needle. The CSF samples were frozen immediately on dry ice and kept at − 80 °C until use.

### Human brain extract preparation

The post-mortem human SN tissue (0.1 g of fresh tissue) was homogenized in ice-cold lysis buffer containing 10 mM Tris HCl, 1 mM EDTA, 300 mM KCl (pH 7.4) and a protease inhibitor cocktail (Roche Molecular Systems, Pleasanton, CA) using a Polytron for 3 × 10 s on ice. The homogenate was centrifuged at 1000 × g for 10 min at 4 °C. The resulting supernatant was centrifuged at 12,000 × g for 30 min at 4 °C. The resulting pellet was dispersed in 50 mM Tris HCl (pH 7.4) and 10 mM MgCl_2_, washed, and resuspended in the same medium. Protein concentration was determined using the bicinchoninic acid (BCA) protein assay kit (ThermoFisher Scientific) and 50–80 μg of protein was used for immunoblotting.

### Gel electrophoresis and immunoblotting

Brain proteins were separated by sodium dodecylsulfate-polyacrylamide gel electrophoresis (SDS-PAGE) and then transferred to polyvinylidene difluoride membranes using the Trans-Blot®Turbo™ transfer system (Bio-Rad) at 25 mV for 30 min. Non-specific binding was blocked by incubation with 5% milk in PBS containing 0.05% Tween for 1 h at room temperature. After washing, the membranes were incubated at 4 °C overnight with the indicated primary antibody in PBS containing 5% milk and 0.05% Tween. Then the membranes were incubated for 1 h with the appropriate horseradish peroxidase (HRP)-conjugated secondary antibody and the immune-complexes were revealed with a chemiluminescence reagent (ECL, Amersham, GE Healthcare, Buckinghamshire, UK) using an Amersham Imager 600 (GE Healthcare Europe GmbH, Barcelona, Spain). The gel protein loading was assessed by using the rabbit anti-α-actinin polyclonal antibody (1 μg/ml). Protein bands were quantified by densitometric scanning and analysed with the Image J program (National Institutes of Health, Bethesda, MA).

### RNA purification and reverse transcription

RNA was extracted from human post-mortem SN, FC or EC tissues using the RNeasy Lipid Tissue Mini Kit (Qiagen, Hilden, Germany) following the manufacturer’s protocol, in combination with DNase digestion to avoid extraction and later amplification of genomic DNA. The concentration of each sample was determined by absorbance at 260 nm using a NanoDrop 2000 spectrophotometer (ThermoFisher Scientific). RNA integrity was determined using the Agilent 2100 BioAnalyzer (Agilent, Santa Clara, CA). Special care was taken to assess pre-mortem and post-mortem factors, which may interfere with RNA processing [32]. Bivariate analyses were carried out to detect the association of our variables with potential confounding factors (age, PMD and RIN) using Spearman or Pearson correlations for quantitative variables. PMD had no effect on RIN values in the present post-mortem samples used (data not shown).

Reverse transcription of the RNA samples was carried out with the High-Capacity cDNA Archive kit (Applied Biosystems, Foster City, CA) and the Gene Amp® 9700 PCR System thermocycler (Applied Biosystems). A parallel reaction for an RNA sample was processed in the absence of reverse transcriptase to rule out DNA contamination.

### qPCR

Real-time quantitative PCR (RT-qPCR) assays were conducted in duplicate on 1000 ng of cDNA samples obtained from the reverse-transcription reaction, diluted at 1:20 in 384-well optical plates (Kisker Biotech, Steinfurt, GE), using the ABI Prism 7900 HT Sequence Detection System (Applied Biosystems). Parallel amplification reactions were carried out using 20× TaqMan Gene Expression Assays and 2× TaqMan Universal PCR Master Mix (Applied Biosystems). β-Glucuronidase (*GUS-β;* Ref: Hs00939627_m1), X-prolyl aminopeptidase (aminopeptidase P) 1 (*XPNPEP1*, Ref: Hs00958026_m1), alanyl-transfer RNA synthase (*AARS*, Ref: Hs00609836_m1) and hypoxanthine-guanine phosphoribosyltransferase (*HPRT*, ref: Hs02800695_m1) were used as housekeeping genes for normalization of *GPR37* (Ref: Hs00173744_m1) expression. The selection of these housekeeping genes was based on previous data showing low vulnerability in the brains of several human neurodegenerative diseases [32, 33]. The RT-qPCR reactions were performed at 50 °C for 2 min, 95 °C for 10 min, and 40 cycles of 95 °C for 15 s and 60 °C for 1 min. TaqMan PCR data were captured using the Sequence Detection Software (SDS version 2.2, Applied Biosystems). Subsequently, the threshold cycle (CT) data for each sample were analyzed with the ΔΔCT method. First, ΔCT values were calculated as the normalized CT values for each target gene in relation to the mean values of *GUS-β*, *XPNPEP1*, *AARS* and *HPRT*. Second, the ΔΔCT values were obtained by the ΔCT of each sample minus the mean ΔCT of the population of control samples (calibrator samples). The fold-change was determined using the eq. 2^-ΔΔCT^.

### Generation of the ecto-GPR37 constructs

The human and mouse GPR37 were tagged at its N-terminal tail with either NanoLuc (NL) luciferase or the O^6^-alkylguanine-DNA alkyltransferase (AGT) to allow further luminescence or AGT substrate (i.e. SNAP tag) detection, respectively. In addition, an haemagglutinin (HA) epitope was also inserted to allow HA-based purification from cells expressing the corresponding constructs. First, the HA-epitope cDNA sequence was inserted into the pNFL1-secN plasmid (Promega, Madison, WI) using the QuikChange II Site-Directed Mutagenesis Kit (Stratagene, San Diego, CA) and the following primer pairs: FNLHA: (5′-**GCCGGATTATGCG**GCGATCGCTTCCGAATTCAGAGCTCAAC-3′) and RNLHA (5′-**ACATCATACGGATA**GCCGCTCGAGCCCGCCAG-3′). Subsequently, the cDNA encoding mouse GPR37 was amplified by PCR using the primers FNL*Eco*RVGPR37 (5′-AAACA**GATATC**TGCTGCCCTGAGCTTTGTCCCT-3′) and R*Not*INLmGPR37 (5′-AA**GCGGCCGC**TCAGCAATGAGTCCCCAC-3′). The amplified mouse GPR37 insert was then cloned into the *Xba*I/*Not*I sites of pNFL1-secN-HA plasmid, thus providing the pNFL-HA-mGPR37^NL^ construct. Similarly, the human GPR37 was amplified using the primers FNL*Xba*IhGPR37 (5′-AAACA**TCTAGA**GCCCTCGGGGTCGCCCCT-3′) and RNL*Not*IhGPR37 (5′-CGA**GCGGCCGC**TCAGCAATGAGTTCCGACAG-3′) and the amplified human GPR37 insert was cloned into the *Eco*RV/*Not*I sites of pNFL1-secN-HA plasmid to provide the pNFL-HA-hGPR37^NL^ construct. Finally, the pRK5-HA-mGPR37^SNAP^ and pRK5-HA-hGPR37^SNAP^ constructs were obtained by amplifying mouse GPR37 with primers FpRK5*Eco*RVmGPR37 (5′-AAACA**GATATC**GCTGCCCTGAGCTTTGTCCCT-3′) and R*Not*INLmGPR37 (5′-AA**GCGGCCGC**TCAGCAATGAGTCCCCAC-3′), and human GPR37 with primers FpRK5*Xho*ImGPR37 (5′-AAACA**CTCGAG**GCCCTCGGGGTCGCCCCT-3′) and RNL*Not*ImGPR37 (5′-CGA**GCGGCCGC**TCAGCAATGAGTTCCGACAG-3′). The amplified mouse or human GPR37 insert was then cloned into the *Eco*RV/*Not*I and *Xho*I/*Not*I sites of pRK5-HA plasmid, thus providing the pRK5-HA-mGPR37^SNAP^ and the pRK5-HA-hGPR37^SNAP^ constructs, respectively. All the constructs were verified by DNA sequencing.

### Cell culture and ecto-GPR37 recombinant protein production

Human embryonic kidney (HEK)-293 T cells (American Type Tissue Culture, Manassas, VA) were grown in Dulbecco’s modified Eagle’s medium (DMEM) (Sigma-Aldrich) supplemented with 1 mM sodium pyruvate, 2 mM *L*-glutamine, 100 U/ml streptomycin, 100 mg/ml penicillin and 5% (*v*/*v*) fetal bovine serum at 37 °C in an atmosphere of 5% CO_2_. Cells were passaged at 80–90% confluency.

HEK-293 T cells grown on 10-cm culture dishes were transiently transfected with the pNFL-HA-mGPR37^NL^, pNFL-HA-hGPR37^NL^, pRK5-HA-mGPR37^SNAP^ or pRK5-HA-hGPR37^SNAP^ construct using the polyethylenimine method [34]. After 24 h, the culture medium was replaced with DMEM and the supernatant was collected 48 h and 72 h after transfection. For purification of the expressed recombinant proteins, the supernatant was first centrifuged at 1000×g for 5 min to remove cell debris, and then concentrated and dialysed in Tris-buffered saline (TBS, 50 mM Tris-Cl, 150 mM NaCl, pH 7.5) using an Amicon-Ultra-15 10 K falcon tube (Merck, Darmstadt, Germany) upon continuous centrifugation at 3000×g at 4 °C until red phenol was not observed. Subsequently, the supernatant was incubated with Pierce™ anti-HA agarose (1 ml, ThermoFisher Scientific, Inc., Rockford, IL) in an end-over-end rotator overnight at 4 °C. After packing and washing the resin with TBS, the recombinant proteins were eluted with glycine-HCl pH 2.5, collected in 1 ml fractions and rapidly neutralized with Tris-base (pH 8.8). Fractions containing the purified recombinant proteins (i.e. mGPR37^NL^, hGPR37^NL^, mGPR37^SNAP^ or hGPR37^SNAP^) were analysed by immunoblot and the luminescence of NL-containing constructs was determined. Finally, the purified proteins were concentrated 40-fold using an Amicon-Ultra-4 3 K falcon tube (Merck) upon continuous centrifugation at 4000×g at 15 °C, and then diluted (1:100) in a buffer containing 10 mM Tris-HCl and 150 mM NaCl, pH 7.4 in a total volume of 400 μl, before being quantified by Micro BCA™ Protein Assay Kit (ThermoFisher Scientific) and Flamingo (Bio-Rad, Hercules, CA) fluorescent protein gel staining.

### NL-based enzyme-linked immunosorbent assay (ELISA) for Ecto-GPR37

CSF ecto-GPR37 was measured with the home-made competitive ELISA method. In brief, streptavidine-coated 96-well white plates (ThermoFisher Scientific) were blocked with serum-free DMEM medium (100 μl/well) for 1 h at 22 °C. After washing (3 × 1 min) with ELISA buffer (25 mM Tris base, 150 mM NaCl, 0.1% BSA, 0.05% Tween-20, pH 7.2), the wells were coated with 100 μl of anti-rabbit IgG-biotin antibody (10 μg/ml, Sigma Aldrich) in the ELISA buffer for 2 h at 22 °C. Subsequently, the plates were washed with the ELISA buffer (3 × 1 min) and incubated with rabbit anti-GPR37 (mouse) or rabbit anti-GPR37 (human) antibody (1 μg/ml in ELISA buffer) for 2 h at 22 °C. Then, 3 μl of either mouse or human CSF was added to each well containing 47 μl of ELISA buffer and incubated overnight at 4 °C in a humid chamber. Next day, the plates were washed (3 times, 1 min each) with ELISA buffer and incubated with 50 μl of purified ecto-GPR37^NL^ (mouse) or ecto-GPR37^NL^ (human) (50.000 relative light units [RLU]/μl in ELISA buffer) for 1 h at 22 °C in the dark. Finally, the plates were washed (3 × 1 min) with ELISA buffer and 90 μl of ELISA buffer was added to each well before incorporating 10 μl of ELISA buffer solution containing 10 μM coelenterazine 400a (NanoLight Technologies, Pinetop, AZ). After 1-min incubation, the end-point luminescence was determined using a CLARIOstar Optima plate-reader (BMG Labtech GmbH, Ortenberg, Germany) and the output luminescence was reported as the integral RLU. In addition, a standard curve was generated using known concentrations of either mouse or human ecto-GPR37^SNAP^ in each experiment.

### α-Synuclein ELISA

The α-synuclein concentration was measured using the LEGEND MAX™ Human α-Synuclein ELISA Kit (844101, BioLegend, San Diego, CA) according to the manufacturer’s instructions. Briefly, a microtiter plate pre-coated with the capture antibody was washed 4 times, added with 20 μl of CSF at 1:10 dilution in reagent buffer, and incubated overnight at 4 °C with shaking. Next day, the plate was washed 4 times, followed by addition of the biotinylated detection antibody to each well and incubation for 2 h at room temperature (RT). The unbound detection antibody was removed by 4 washes. The streptavidin-HRP conjugate was added to each well, incubated for 1 h at RT and then the plate was washed 4 times to remove the remaining conjugate. Finally, chemiluminescent substrate was added to each well and signals were read using a Tecan Spark 10 M plate reader (Tecan Nordic AB, Stockholm, Sweeden).

### Statistical analysis

Data are presented as mean ± standard error of mean (SEM) and were analysed by Student’s *t* test or one-way analysis of variance (ANOVA) with Dunnett’s *post-hoc* test using GraphPad Prism 6.01 (San Diego, CA). *P* < 0.05 was considered as statistically significant. The distribution of CSF ecto-GPR37 did not follow Gaussian distribution (Shapiro-Wilk normality test, *P* < 0.05), so the data were analysed with non-parametric Mann-Whitney U-tests. The diagnostic accuracy by CSF ecto-GPR37 levels was assessed using the receiver operating characteristic (ROC) curve analysis. The correlation coefficients (rho) were calculated using the Spearman two-tailed correlation test.

## Results

### GPR37 density in post-mortem SN from NC and PD subjects

**Fig. 1:**
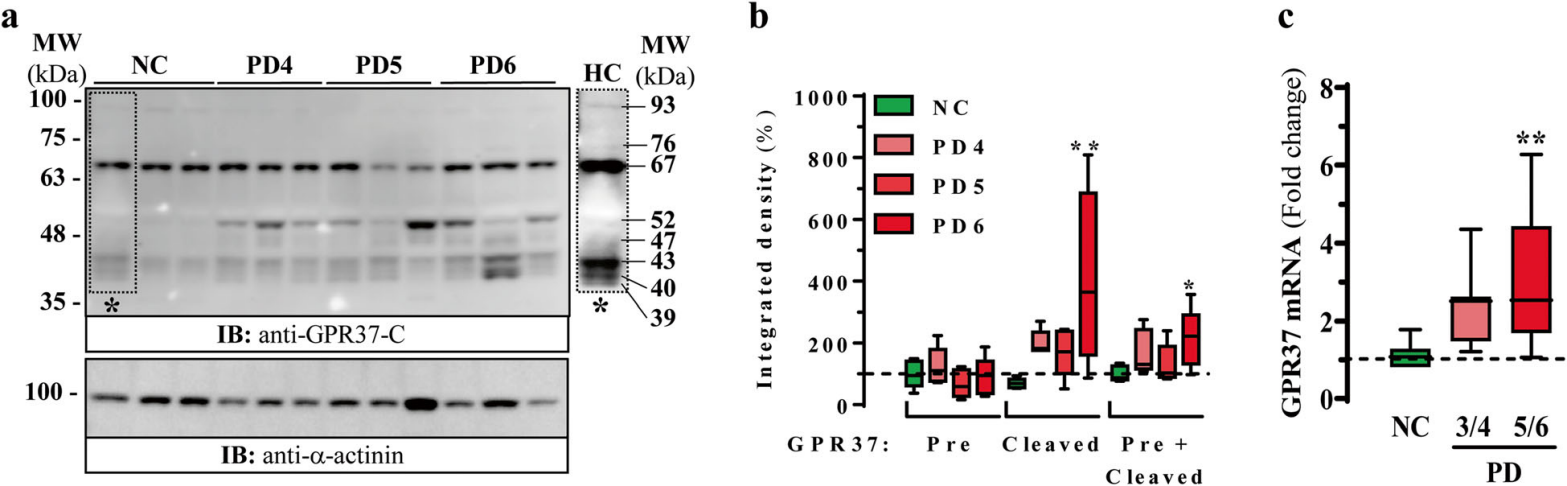
GPR37 protein density and mRNA levels in post-mortem brain tissues from neurological controls (NCs) and Parkinson’s disease (PD) subjects. **a** Immunoblots showing the expression of GPR37 in the SN of NC and PD subjects (Braak PD stage 4, Braak PD 5 and Braak PD 6). Extracts from the human post-mortem SN were analysed by SDS-PAGE and immunoblotted using rabbit anti-GPR37-C and rabbit anti-α-actinin antibodies. The right dashed outlined area (*) is shown with enhanced contrast and corresponds to lane 1 (NC). The different GPR37 forms included precursor (70 kDa), full-length mature (93 kDa) and GPR37 N-terminus-cleaved forms (76 kDa, 52 kDa, 47 kDa, 43 kDa and 39–40 kDa). **b** Relative quantification of GPR37 protein density in the SN. The immunoblots corresponding to the precursor (Pre) and cleaved GPR37 forms from the NC (*n* = 5), Braak PD stage 4 (*n* = 5), Braak PD stage 5 (*n* = 5) and Braak PD stage 6 (*n* = 5) subjects were quantified by densitometric scanning. Values were normalized to α-actinin in each lane to correct for protein loading. Results are expressed as the percentage (mean ± SEM) of the NC. *P < 0.05, **P < 0.01 vs NC, one-way ANOVA followed by Dunnett’s *post-hoc* test. **c** RT-qPCR assessment of GPR37 mRNA expression in post-mortem SN from NCs (*n* = 10), Braak PD stage 3/4 (*n* = 7) and Braak PD stage 5/6 (*n* = 17) subjects. Mean values of the housekeeping genes (*GUS-B*, *XPNPEP1*, *AARS* and *HPRT*) were used to normalize samples. Results are expressed as fold-change (mean ± SEM) of the NCs. **P < 0.01 vs NC, one-way ANOVA followed by Dunnett’s *post-hoc* test

Metalloprotease-mediated GPR37 N-terminus cleavage leads to ectodomain shedding in a heterologous expression system [13]. This posttranslational modification, together with N-glycosylation, generates a heterogenous pattern of GPR37 species that can be detected by immunoblotting, namely the precursor, the full-length mature and some N-terminus-cleaved receptor forms [13]. Here, we first assessed GPR37 density in human SN by immunoblotting using a specific antibody against human GPR37 C-terminal domain (anti-GPR37-C) [18]. The immunoblotting results revealed a wide-range of GPR37 protein bands (Fig. 1a) compatible with the putative precursor (67 kDa), the full-length mature (93 kDa) and several GPR37 N-terminus-cleaved forms (76 kDa, 52 kDa, 47 kDa, 43 kDa and 39–40 kDa) already described [13]. This is consistent with a recent report of band pattern using a commercial anti-GPR37 C-terminal-domain antibody (RRID: AB_2799658) (https://www.cellsignal.com/products/primary-antibodies/gpr37-d4c8h-rabbit-mab/64407). These results indicate that in human native tissues (e.g. SN), GPR37 may be N-terminally processed, thus suggesting the existence of a heterogenous pattern of GPR37 species.

GPR37 accumulates in SN LBs of PD patients [35], thus we next assessed the density of the different N-terminally processed forms of GPR37 in SN of PD subjects. The GPR37 precursor density did not increase throughout the different Braak PD stages assayed, but the N-terminal-cleaved forms were significantly (*P* = 0.0057) increased at Braak PD stage 6 (Fig. 1b), rendering an average of 214% ± 42% increase of density (*P* = 0.0462) when all the GPR37 forms (i.e. precursor plus N-terminus cleaved forms) were considered (Fig. 1b). These results indicated an overall increased density of GPR37 in SN of PD subjects, particular the N-terminal-cleaved forms of the receptor. Moreover, RT-qPCR analysis showed a significant (*P* = 0.0011) 3.1-fold increase in GPR37 mRNA expression in SN of Braak PD stage 5/6 subjects as compared to the NC subjects (Fig. 1c). Collectively, these results confirmed an upregulation of GPR37 expression in sporadic PD and a concomitant increase in N-terminally cleaved forms of GPR37.

### Ecto-GPR37 in CSF

**Fig. 2:**
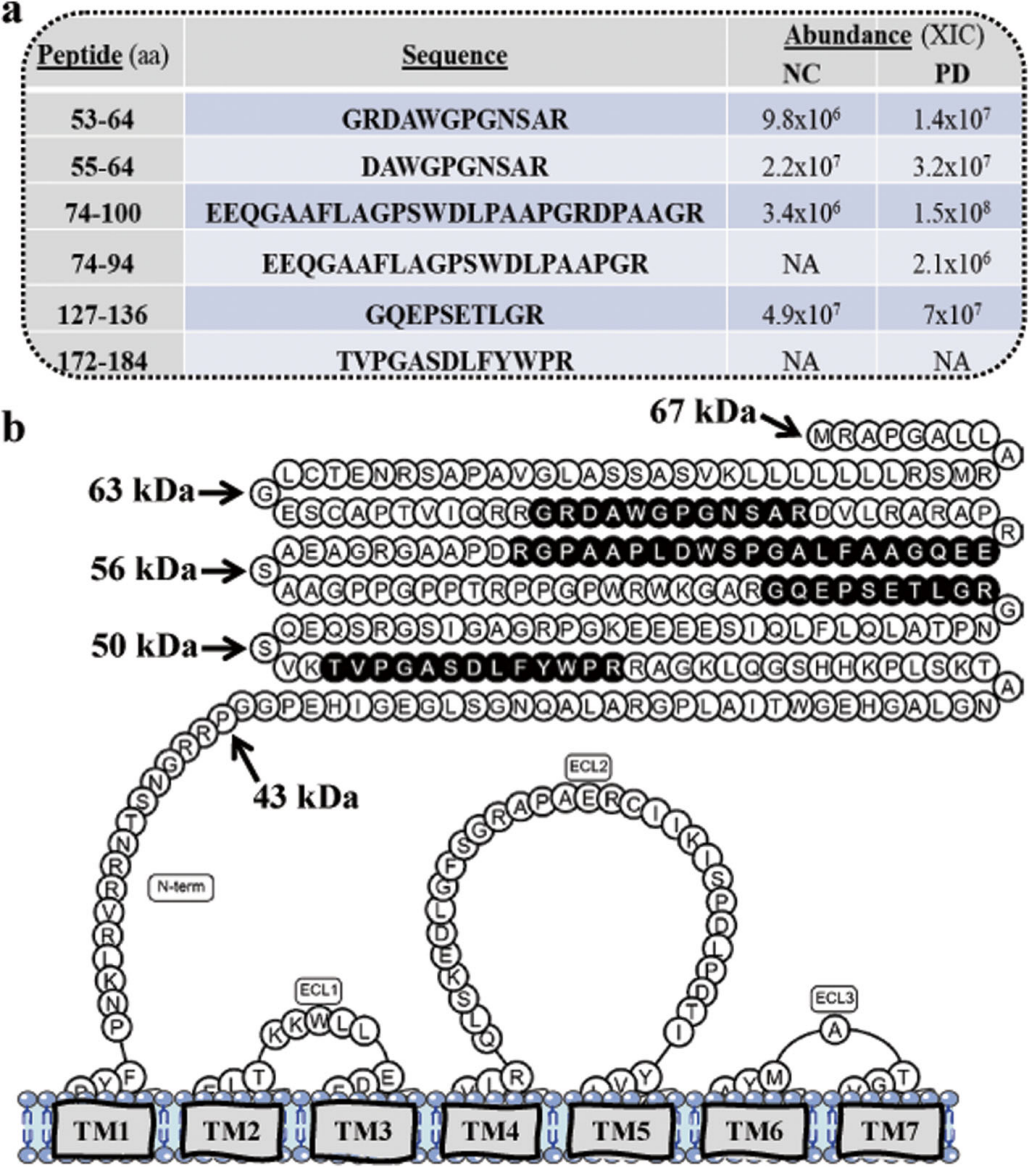
Ecto-GPR37 peptides detected in human CSF. **a** Mass spectrometric results (*see* Supplementary Methods) of human ecto-GPR37 peptides immunoprecipitated from the pooled CSF of NC (*n* = 10) or PD subjects (*n* = 10). The detected peptide sequences in the CSF of NC and PD are indicated (M + H^+^) and their estimated abundance was expressed as the area from the extracted ion chromatograms (XIC) of the peptide chromatographic peak. *NA*, not quantified. **b** Schematic representation showing the precise location of the mass spectrometry-detected ecto-GPR37 peptides (black circles) within the N-terminal domain (N-term) of the receptor. The theoretical molecular weight (kDa) based on the amino acid sequence of human GPR37 is indicated (arrows). The seven transmembrane (TM) domains of the receptor embedded within the membrane are highlighted in grey. The three extracellular loops (ECL) are also indicated. The GPR37 scheme was made using the GPCRdb resource [36]

To test if GPR37 shedding also occurs in native tissue, we monitored GPR37 in vivo shedding by detecting the excised N-terminus of GPR37 (i.e. ecto-GPR37) in the CSF. To confirm the identity of ecto-GPR37 in CSF, we immunoprecipitated ecto-GPR37 using the anti-GPR37-N antibody and the isolated peptides were analysed by liquid chromatography coupled to mass spectrometry (*see* Supplementary Methods). Six unique peptides corresponding to the N-terminal domain of the human GPR37 in pooled CSF samples from NC and PD patients were detected (Fig. 2). Ecto-GPR37 peptides, especially the EEQGAAFLAGPSWDLPAAPGRDPAAGR peptide, showed an overall increased abundance in CSF from PD patients when compared to that from NC subjects (Fig. 2a). Indeed, these results were consistent with the increased density of N-terminally cleaved forms of the receptor shown in the immunoblot analysis of post-mortem SN from PD subjects (Fig. 1a and b).

**Fig. 3:**
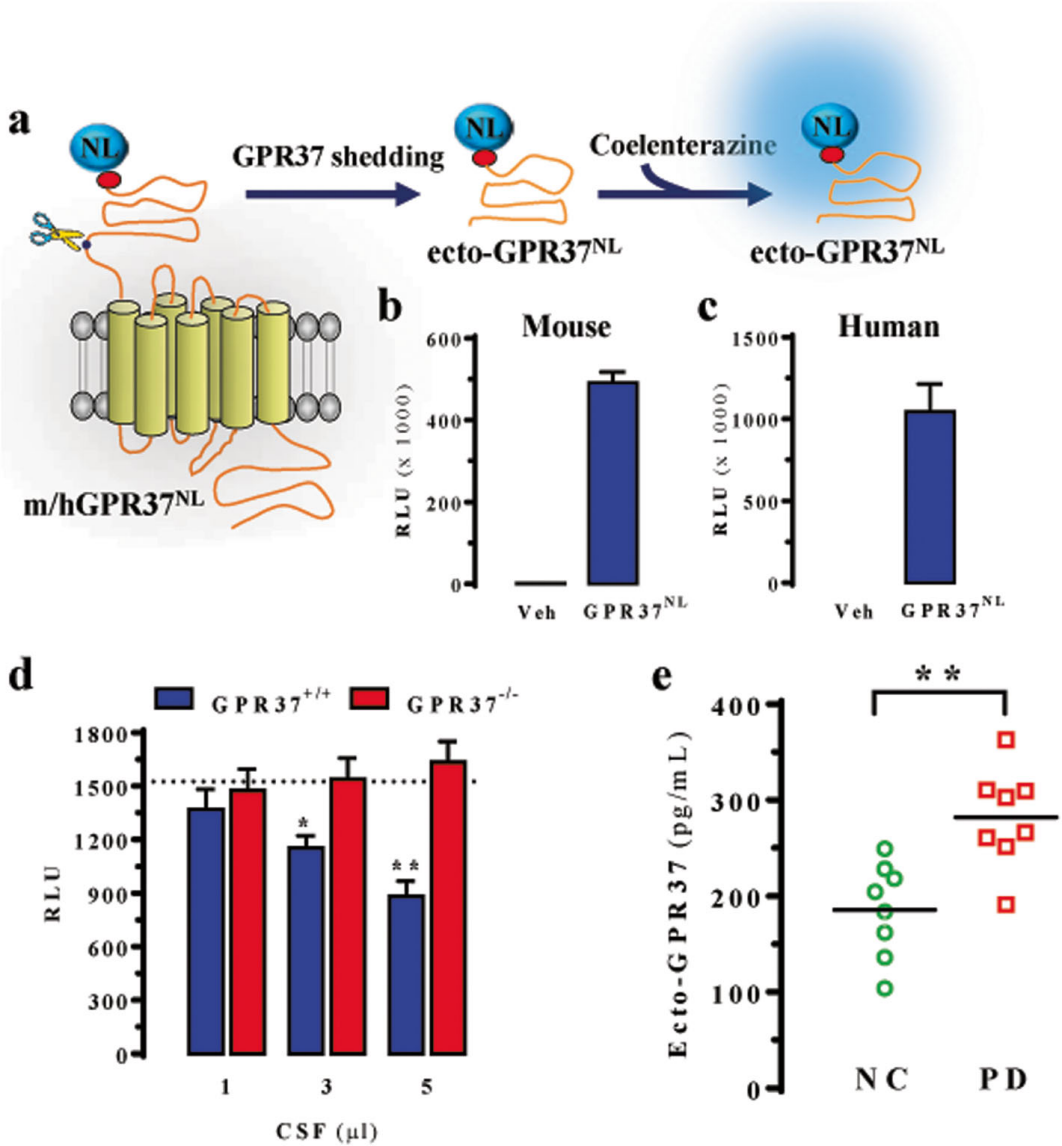
Detection of ecto-GPR37 by the NanoLuc (NL)-based immunoassay. **a** Schematic representation showing the putative metalloproteinase (scissors)-mediated cleavage and shedding of the GPR37 N-terminal domain (i.e. ecto-GPR37) in a GPR37 construct containing the HA (filled red circle) and NL tags at its N-terminus (i.e. ecto-GPR37^NL^). Upon expression in HEK-293 cells, the GPR37^NL^ ectodomain was released by shedding and detected in the culture medium by monitoring its NL enzymatic activity after incubation of the growth medium of cells transfected with mock (Veh) or mouse (**b**)/human (**c**) GPR37^NL^ with coelenterazine 400a and determination of the luminescence (RLU, relative light units). **d** Assessment of ecto-GPR37 in 1 μl, 3 μl and 5 μl of cerebrospinal fluid of GPR37^+/+^ and GPR37^−/−^ mice. The purified mouse ecto-GPR37^NL^ was used as a tracer in the competitive ELISA. Results are expressed as RLU (mean ± SD, *n* = 3). Dashed line represents the RLU_max_ (i.e. RLU in the absence of CSF). *P < 0.05 and **P < 0.01 vs RLU_max_, one-way ANOVA followed by Dunnett’s *post-hoc* test. **e** Assessment of ecto-GPR37 in post-mortem CSF from NC (*n* = 8) and PD (*n* = 8) subjects from Bellvitge Hospital using the GPR37-NL-based ELISA assay. **P < 0.01, Student’s *t*-test

Subsequently, we developed an in-house species-specific NL-based ELISA assay to detect either mouse or human ecto-GPR37 at the extracellular milieu (i.e. CSF). First, we engineered specific mouse or human GPR37 constructs containing HA and NL tags at the N-terminus (Fig. 3a) to allow their purification and further luminescence detection (Fig. 3b and c). The recombinant mouse or human ecto-GPR37^NL^ protein was used as a tracer in an ELISA assay operated with a rabbit anti-mouse/human GPR37-N polyclonal antibody captured to microplates coated with an anti-rabbit antibody. Indeed, the sensitivity of the ELISA assay was previously assessed by performing competition assays with recombinant mouse or human ecto-GPR37^SNAP^ protein (data not shown). Subsequently, we determined the ecto-GPR37 density in mouse and human CSF. First, CSF from wild-type and GPR37^−/−^ mice was assayed with the specific mouse GPR37-NL-based ELISA assay. While significant amount of ecto-GPR37 was detected in the CSF from GPR37^+/+^ mice, which was determined to be 286.8 ± 77.3 pg/ml, ecto-GPR37 was undetectable in all CSF volumes from the GPR37^−/−^ mice assayed (Fig. 3d). These results indicate that the specific mouse ecto-GPR37 ELISA assay unequivocally detected ecto-GPR37 in the mouse CSF.

**Fig. 4:**
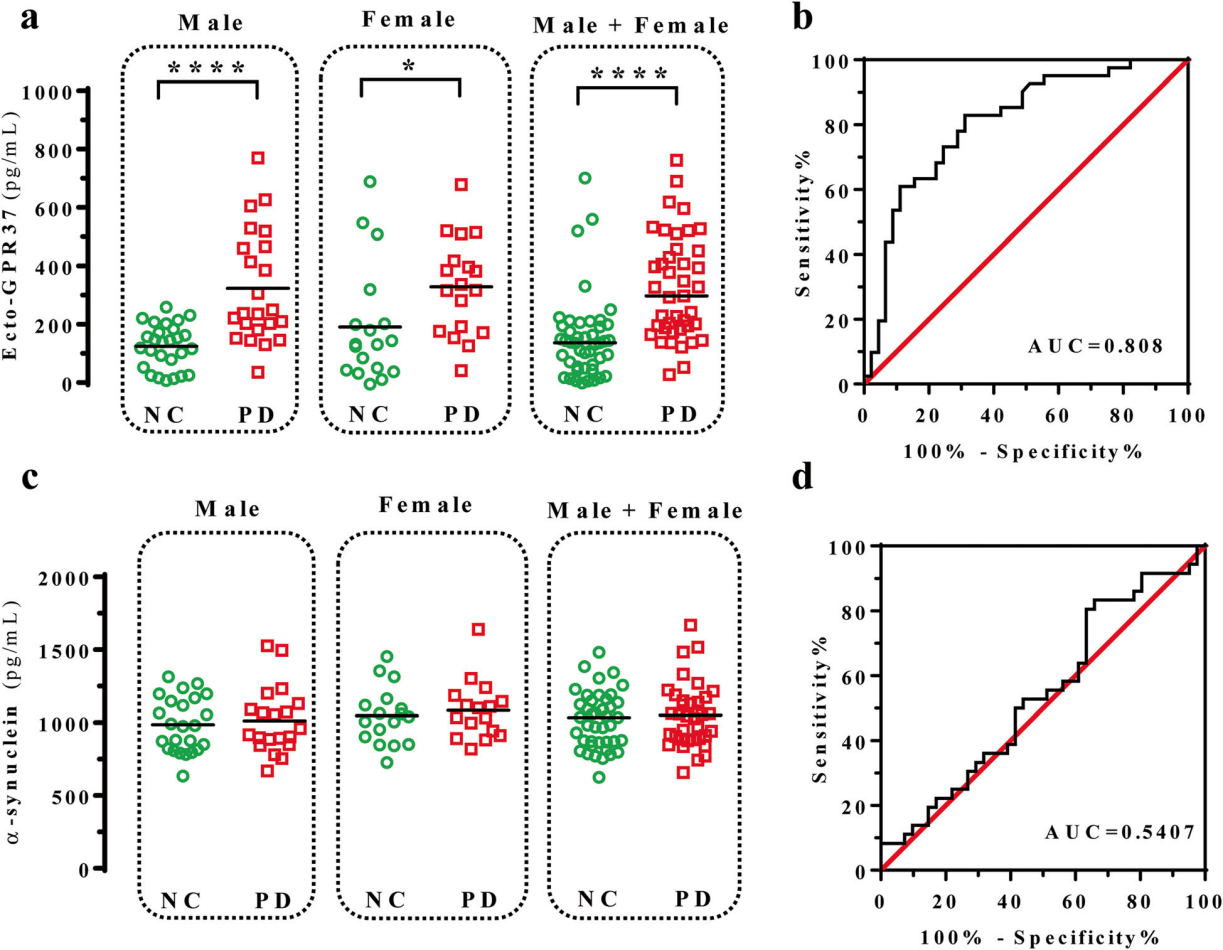
Detection of ecto-GPR37 and α-synuclein in CSF of NC and PD participants from Karolinska Hospital. **a** The CSF ecto-GPR37 level was significantly higher in PD patients than in NCs, irrespective of sex. **b** ROC curve analysis of CSF ecto-GPR37. **c** CSF α-synuclein did not differ between the NC and PD participants. **d** ROC curve analysis of CSF α-synuclein. *P < 0.05, ****P < 0.0001 (Mann-Whitney U-tests)

Next, we analysed post-mortem human CSF from a small cohort (*n* = 8) of NC and PD subjects using the specific human GPR37 NL-based ELISA assay. While the NCs had an average ecto-GPR37 content of 185 ± 17 pg/ml in the CSF, the PD patients had 282 ± 18 pg/ml ecto-GPR37 in the CSF. Thus, ecto-GPR37 was significantly (*P* = 0.019) increased in the CSF of PD patients (Fig. 3e). Subsequently, a larger and different cohort of NC (*n* = 45) and PD (*n* = 41) subjects was assayed. Again, significantly higher levels of CSF ecto-GPR37 were found in the PD subjects (334 ± 28 pg/ml) when compared to the NCs (159 ± 21 pg/ml) (*P* = 0.001) (Fig. 4a). ROC curve analysis demonstrated that the human GPR37 NL-based ELISA assay could differentiate PD subjects (*n* = 41) from NCs (*n* = 45), with the area under the curve (AUC) of 0.808 (0.713–0.900) (*P* < 0.0001) (Fig. 4b). The assay, with a cut-off value of 200 pg/ml, had a sensitivity of 73%, a specificity of 73%, a positive predictive value of 71%, and a negative predictive value of 75% for PD diagnosis (Fig. 4b). In addition, no statistically significant correlations between age/gender and CSF ecto-GPR37 levels were observed in neither the NC nor the PD group (Fig. S1). Also, there were no correlations between the ecto-GPR37 levels in the PD group and the main clinical features monitored (Table 1, Fig. S2).

Since α-synuclein has attracted considerable attention as a potential molecular biomarker for PD, we analysed the α-synuclein content in the CSF of the same PD cohort (Table 1). No significant difference in the total α-synuclein concentration in the CSF was found between the NC and PD subjects (1049 ± 39 pg/ml vs 1061 ± 37 pg/ml, *P* = 0.4692) (Fig. 4c). The corresponding ROC curve analysis showed that the α-synuclein ELISA assay could not discriminate between the NC and PD subjects (AUC = 0.5407, *P* = 0.5402) (Fig. 4d). Overall, the results suggested that the CSF total α-synuclein could not distinguish PD patients from control subjects.

**Fig. 5:**
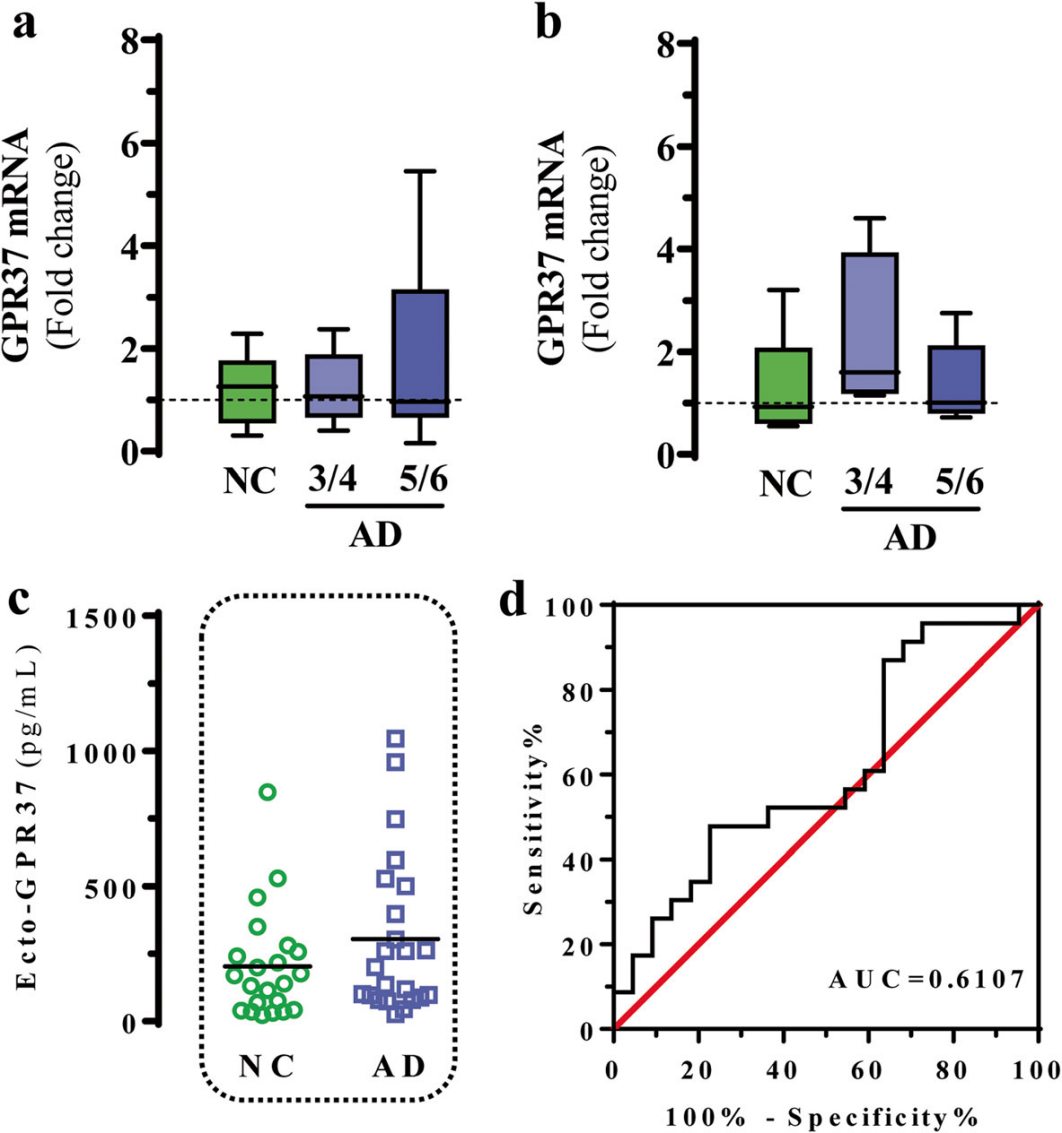
GPR37 mRNA expression and ecto-GPR37 levels in AD. GPR37 mRNA expression in post-mortem frontal cortex (**a**) and entorhinal cortex (**b**) from NCs (panel **a**: *n* = 17; **b**: *n* = 5), Braak AD stage 3/4 (**a**: *n* = 9; **b**: *n* = 4) and Braak AD stage 5/6 (**a**: *n* = 20; **b**: *n* = 5) subjects was assessed by RT-qPCR. Mean values of the housekeeping genes (*GUS-B*, *XPNPEP1*, *AARS* and *HPRT*) were used to normalize samples. Results are expressed as fold-change (mean ± SEM) of the NCs. **c** Detection of ecto-GPR37 in the CSF from NC (*n* = 22) and AD (*n* = 23) participants  recruited at the University Medical School of Göttingen. **d** ROC curve analysis of CSF ecto-GPR37

Last, we assessed whether the CSF ecto-GPR37 had a specific role in the diagnosis of AD, the most common neurodegenerative disorder. To this end, we first evaluated GPR37 mRNA expression levels in the FC and EC from NC and Braak AD stage 3/4 and 5/6 subjects (AD3/4 and AD5/6, respectively) by RT-qPCR. There were no significant changes in GPR37 mRNA expression in either FC or EC among NC, AD3/4 and AD5/6 subjects (Fig. 5a and b). Subsequently, we evaluated the ecto-GPR37 content in the CSF from NC and AD patients using the GPR37 NL-based ELISA assay, and found no significant difference between the NC (203 ± 42 pg/ml) and AD (304 ± 61 pg/ml) subjects (*P* = 0.1266; Fig. 5c). The corresponding ROC curve analysis showed that the human GPR37 NL-based ELISA assay could not discriminate between the NC and AD subjects (AUC = 0.6107, *P* = 0.2036) (Fig. 5d). Although PD and AD pathologies share impaired biological processes, our results suggest a differential molecular mechanism of GPR37 expression and CSF ecto-GPR37 levels between AD and PD. Thus, further studies are required to characterize GPR37 biology in AD.

## Discussion

Although GPR37 has been postulated to be the receptor for the neuroprotective and glioprotective factors prosaptide and prosaposin [37, 38], the precise physiological function of the receptor remains elusive. Recent studies have reported some alternative functions of GPR37, such as the chaperone-like activity that controls the cell surface targeting and function of adenosine and dopamine receptors [18, 39], and participation in the Wnt co-receptor LRP6 maturation [40]. Remarkably, it has been demonstrated that the GPR37 N-terminus is processed by MPs, leading to the generation of different GPR37 N-terminally cleaved forms [13]. While frequently described in the processing of single-pass membrane-anchored proteins, the MP-mediated shedding is less common in post-translational modification of GPCRs [41]. The N-terminal ectodomain of GPR37 has been found to be susceptible to the MP-mediated cleavage, which occurs constitutively in the absence of any external stimulation and leads to the shedding of the receptor N-terminus (i.e. ecto-GPR37). Interestingly, although the GPR37 precursors are efficiently converted into the mature receptor forms (i.e. fully glycosylated forms) and targeted to the plasma membrane, by far most cell surface receptors exist in a cleaved form under steady-state homeostatic conditions. Therefore, it can be speculated that in neurodegenerative conditions which develop with GPR37 overexpression and/or MP alterations, as in PD [14], the ecto-GPR37 levels may mirror the neural damage during disease progression.

In the present study, the presence of ecto-GPR37 peptides in human CSF was first revealed by mass spectrometry. Interestingly, all the ecto-GPR37 peptides found in the CSF of NC subjects (Fig. 2b) corresponded to the N-terminal amino acid sequences upstream the most abundant cleaved form of the receptor (i.e. 43 kDa) detected in our immunoblot experiments. This observation was compatible with the occurrence of constitutive shedding of the receptor in vivo, thus reinforcing the biological relevance of this process for GPR37. In addition, the immunoblotting detection of GPR37 in PD SN revealed upregulation of a prominent 52-kDa receptor form, which correlated with the increased CSF density of upstream N-terminal peptides, especially the EEQGAAFLAGPSWDLPAAPGRDPAAGR and GRDAWGPGNSAR peptides. The precise molecular mechanisms underlying the differential GPR37 shedding in PD require further investigation.

The human GPR37 NL-based ELISA assay allowed reproducible measurement of ecto-GPR37 levels in CSF samples. Here, we found significantly higher levels of ecto-GPR37 in the CSF of PD patients compared to that in the NCs in two independent clinical cohorts, consistent with the higher density of CSF ecto-GPR37 peptides revealed by the proteomic analysis. Interestingly, our findings showed that the CSF ecto-GPR37 achieved diagnostic performance when the CSF samples from the PD patients were analyzed and compared to the NC subjects. The finding of no correlations between clinical ratings and the CSF content of ecto-GPR37 in the PD patients may be related to the fact that this part of the study was underpowered. The lack of correlation with the UPDRS motor scores may be because that the clinical ratings were performed when the patients were ON, i.e. they took their daily treatments before the visit to the clinics. In addition, here we found no alterations of the CSF total α-synuclein in the same PD cohort, which was consistent with previous reports [42–47]. However, there were also studies reporting lower levels of total α-synuclein in the CSF of PD subjects [48]. Other studies have found that the total CSF α-synuclein in PD overlaps with that found in different diseases, thus being not possible to discriminate between patients with synucleinopathies and cognitively normal subjects suffering from other non-neurodegenerative diseases [43]. Finally, two meta-analytic reviews of the existing data revealed that the CSF total α-synuclein is lower in PD patients compared to controls [44, 45]. The sensitivity and specificity for distinguishing PD from controls were 0.72 and 0.65 [44] or 0.88 and 0.4 [45], respectively, and the ROC curve showed an AUC of 0.73 [45]. Thus, while the total CSF α-synuclein by itself shows a low diagnostic accuracy and does not seem to be a reliable PD diagnostic marker, it may provide a higher accuracy in PD diagnosis when combined with other biomarkers [4, 5]. Instead, the real-time quaking-induced conversion (RT-QuIC) assay detected α-synuclein aggregation in PD CSF with a sensitivity of 95% and a specificity of 100% when compared to control CSF [46]. However, RT-QuIC is time-consuming (several days) and cumbersome to perform. In addition to the CSF biomarkers, DatScan SPECT imaging is clinically used to aid PD diagnosis, and has an AUC value of 0.92–0.96 in distinguishing PD from controls [47]. However, the DatScan SPECT is expensive and, from the pathological perspective, it does not perform well to predict the number of nigral neurons in PD [48]. Therefore, there is still a need for novel fluid biomarkers of PD that are inexpensive and easy to perform.

Interestingly, here we found no significant increase of CSF ecto-GPR37 in AD patients compared to the NCs. AD is pathologically characterized by the accumulation of Aβ and pathologically modified tau proteins to form senile plaques and NFTs, respectively [49]. In addition, AD patients often present with accumulation of other proteins including α-synuclein and TDP-43, which may play a pathogenic role in AD and/or reflect the diversity of AD pathophysiology. Thus, biomarkers based on these proteins are widely considered as useful diagnostic markers of AD. The results of CSF ecto-GPR37 in AD suggest different pathological participation of GPR37 in PD versus AD. The differential diagnostic value of GPR37 in PD and AD may be useful for discriminating combined neurodegenerative pathologies, thus allowing correct stratification of these brain disorders, especially at early stage. Further longitudinal studies combining CSF ecto-GPR37 with reliable biomarkers for neurodegenerative diseases are needed. In addition, it would be necessary to explore whether the CSF ecto-GPR37 can be used for the differential diagnosis between PD and atypical parkinsonism, including LB dementia, multiple system atrophy, progressive supranuclear palsy and corticobasal syndrome. Moreover, future studies are needed to elucidate whether the CSF ecto-GPR37 can assist in discriminating the PD patients who will develop a certain phenotype, e.g. PD dementia, and/or those who will have a rapid disease progression.

## Conclusion

In this study, we reported, for the first time, that significantly higher levels of ecto-GPR37 were detectable in the CSF of PD patients. In addition, the CSF ecto-GPR37 achieved good diagnostic performance for PD, in contrast to the total α-synuclein performance. Interestingly, the ecto-GPR37 levels were not increased in the CSF of AD subjects. Thus, our study provides strong evidence supporting CSF ecto-GPR37 as a promising biomarker of PD. Hence, the CSF ecto-GPR37 levels may evolve into a measurement of PD neuropathology, complementing the existing biomarkers. Future studies should be performed to explore the potential use of ecto-GPR37 in combination with other neurodegeneration-related biomarkers in clinical research, trials and practice.

## Supplementary Information

**Additional file 1. Supplementary Methods. Supplementary Figure S1.** Correlations of CSF ecto-GPR37 levels with age in male and female Parkinson’s disease patients. The correlation coefficients (*r*) was calculated using the Spearman two-tailed correlation test. **Supplementary Figure S2.** Correlations of CSF ecto-GPR37 levels with disease duration (a), Unified Parkinson’s Disease Scale (UPDRS, *n* = 26) total (b) and part 3 (c), Hoehn & Yahr (H&Y) scale (*n* = 40) (d), Montreal Cognitive Assessment scores (MoCA, *n* = 28) evaluating the cognitive impairment (e), Hospital Anxiety and Depression scale (HADS, *n* = 26) (f) and *L*-dopa equivalent doses (LEDs, *n* = 28) (g). The correlation coefficients (*r*) was calculated using the Spearman two-tailed correlation test.

## Data Availability

The data that support the findings of this study are available from the corresponding author upon reasonable request.

## Abbreviations

PD: Parkinson’s disease
AD: Alzheimer’s disease
NC: Neurological control
CSF: Cerebrospinal fluid
ELISA: Enzyme-linked immunosorbent assay
GPCR: G protein-coupled receptor
MP: Metalloproteinase
ecto-GPR37: GPR37 ectodomain
NFT: Neurofibrillary tangle
UPDRS: Unified Parkinson’s disease scale
MoCA: Montreal cognitive assessment
BCA: Bicinchoninic acid
SDS-PAGE: Sodium dodecylsulfate-polyacrylamide gel electrophoresis
HRP: Horseradish peroxidase
RT-qPCR: Real-time quantitative polymerase chain reaction
GUS-β: β-Glucuronidase
XPNPEP1: X-prolyl aminopeptidase P
AARS: Alanyl-transfer RNA synthase
HPRT: Hypoxanthine-guanine phosphoribosyltransferase
AGT: O^6^-alkylguanine-DNA alkyltransferase
HA: Haemagglutinin
HEK: Human embryonic kidney
DMEM: Dulbecco’s modified Eagle’s medium
ROC: Receiver operating characteristic
NL: NanoLuc luciferase
AUC: Area under the curve
