# Supplementary material for: Ecto-GPR37: a potential biomarker for Parkinson’s disease
Source: Transl Neurodegener. 2021 Feb 26;10:8. doi: 10.1186/s40035-021-00232-7 (PMC7908677; doi:10.1186/s40035-021-00232-7)
Supplement: Supplementary file 1 — Additional file 1. Supplementary Methods. Supplementary Figure S1. Correlations of CSF ecto-GPR37 levels with age in male and female Parkinson’s disease patients. The correlation coefficients (r) was calculated using the Spearman two-tailed correlation test. Supplementary Figure S2. Correlations of CSF ecto-GPR37 levels with disease duration (a), Unified Parkinson’s Disease Scale (UPDRS, n = 26) total (b) and part 3 (c), Hoehn & Yahr (H&Y) scale (n = 40) (d), Montreal Cognitive Assessment scores (MoCA, n = 28) evaluating the cognitive impairment (e), Hospital Anxiety and Depression scale (HADS, n = 26) (f) and L-dopa equivalent doses (LEDs, n = 28) (g). The correlation coefficients (r) was calculated using the Spearman two-tailed correlation test. [file 40035_2021_232_MOESM1_ESM.docx]

**Supplementary information**

**Ecto-GPR37: a potential biomarker for Parkinson’s disease**

*Xavier Morató^1,2,3^, Paula Garcia-Esparcia^2,4,5^, Josep Argerich^1,2^, Franc Llorens^2,4,5,6^, Inga Zerr^6,7^, Wojciech Paslawski^3^, Eva Borràs^8,9^, Eduard Sabido^8,9^, Ulla E. Petäjä-Repo^10^, Víctor Fernández-Dueñas^1,2^, Isidro Ferrer^2,4,5^, Per Svenningsson^3^ and Francisco Ciruela^1,2^.*

*^1^Pharmacology Unit, Department of Pathology and Experimental Therapeutics, Faculty of Medicine and Health Sciences, Institute of Neurosciences, University of Barcelona, L’Hospitalet de Llobregat, Spain. ^2^Neuroscience Program, Institut d’Investigació Biomèdica de Bellvitge, IDIBELL, L’Hospitalet de Llobregat, Spain. ^3^Section of Neurology, Department of Clinical Neuroscience, Department of Neuroscience, Karolinska Institutet, Stockholm, Sweden. ^4^Neuropathology Unit, Department of Pathology and Experimental Therapeutics, Faculty of Medicine and Health Sciences, Institute of Neurosciences, University of Barcelona, L’Hospitalet de Llobregat, Spain. ^5^CIBERNED, Centro de Investigación Biomédica en Red de Enfermedades Neurodegenerativas, Instituto Carlos III, Spain. ^6^Department of Neurology, Clinical Dementia Center and National Reference Center for CJD Surveillance, University Medical School, Göttingen, Germany.^7^German Center for Neurodegenerative Diseases (DZNE), Göttingen, Germany. ^8^Proteomics Unit, Center for Genomic Regulation, Spain. ^9^Proteomics Unit, Universitat Pompeu Fabra. ^10^Research Unit of Biomedicine, Medical Research Center Oulu, University of Oulu, Oulu, Finland.*

**Supplementary Methods**

**CSF Proteomic analysis**

*Sample preparation*. Ecto-GPR37 was immunoprecipitated from human CSF using the anti-human-GPR37-N polyclonal antibody. To this end, 850 μl of antibody (500 μg/ml) was covalently captured to 300 μl tosylactivated magnetic beads (Dynabeads M-280; ThermoFisher Scientific) at 4°C for 48 h, following manufacturer’s indications. The beads were isolated with a magnet, washed (3 times, 1 min each) with phosphate-buffered saline (PBS; 8.07 mM Na_2_HPO_4_, 1.47 mM KH_2_PO_4_, 137 mM NaCl, 0.27 mM KCl, pH 7.2) containing 0.1% BSA and 0.05% Tween-20 (PBS-T-BSA) and resuspended in 1ml of PBS-T-BSA. Subsequently, 650 μl of pooled CSF from control or PD subjects was immunoprecipitated with 500 μl anti-hGPR37 antibody-coupled magnetic beads overnight at 4°C. Magnetics beads were isolated, the supernatant removed and washed (3 times, 1 min each) with PBS. Bound peptides/proteins were eluted from the beads in 6M Urea / 200mM ammonium bicarbonate. Samples were then reduced with dithiothreitol (30 nmol, 37 ºC, 60 min), alkylated in the dark with iodoacetamide (60 nmol, 25 ºC, 30 min) and diluted to 1M urea with 200 mM ammonium bicarbonate for trypsin digestion (1 µg, 37ºC, 8h, Promega cat # V5113). After digestion, peptide mix was acidified with formic acid and desalted with a MicroSpin C18 column (The Nest Group, Inc, Southborough, MA, USA) prior to LC-MS/MS analysis.

*Chromatographic and mass spectrometric analysis.* Samples were analyzed using a LTQ-Orbitrap Velos Pro mass spectrometer (ThermoFisher Scientific) coupled to an EASY-nLC 1000 (Thermo Fisher Scientific (Proxeon), Odense, Denmark). Peptides were loaded onto the 2-cm Nano Trap column with an inner diameter of 100 μm packed with C18 particles of 5 μm particle size (ThermoFisher Scientific) and were separated by reversed-phase chromatography using a 25-cm column with an inner diameter of 75 μm, packed with 1.9 μm C18 particles (Nikkyo Technos Co., Ltd. Japan). Chromatographic gradients started at 93 % buffer A and 7 % buffer B with a flow rate of 250 nl/min for 5 min and gradually increased 65 % buffer A and 35 % buffer B in 60 min. After each analysis, the column was washed for 15 min with 10% buffer A and 90 % buffer B. Buffer A: 0.1 % formic acid in water. Buffer B: 0.1 % formic acid in acetonitrile.

The mass spectrometer was operated in positive ionization mode with nanospray voltage set at 2.1 kV and source temperature at 300 °C. Ultramark 1621 for the was used for external calibration of the FT mass analyzer prior the analyses, and an internal calibration was performed using the background polysiloxane ion signal at m/z 445.1200. The acquisition was performed in data-dependent acquisition (DDA) mode and full MS scans with 1 micro scans at resolution of 60,000 were used over a mass range of m/z 350-2000 with detection in the Orbitrap. Auto gain control (AGC) was set to 1E6, dynamic exclusion (60 seconds) and charge state filtering disqualifying singly charged peptides was activated. In each cycle of DDA analysis, following each survey scan, the top twenty most intense ions with multiple charged ions above a threshold ion count of 5000 were selected for fragmentation. Fragment ion spectra were produced via collision-induced dissociation (CID) at normalized collision energy of 35 % and they were acquired in the ion trap mass analyzer. AGC was set to 1E4, isolation window of 2.0 m/z, an activation time of 10 ms and a maximum injection time of 100 ms were used. All data were acquired with Xcalibur software v2.2.

Digested bovine serum albumin (cat # P8108S, New England Biolabs, Ipswich, MA, USA) was analyzed between each sample to avoid sample carryover and to assure stability of the instrument and QCloud has been used to control instrument longitudinal performance during the project [1].

*Data Analysis.* Acquired spectra were analyzed using the Proteome Discoverer software suite (v2.0, ThermoFisher Scientific) and the Mascot search engine (v2.5 Matrix Science). The data were searches against a Swiss-Prot human database (as in April 2018, 20797 entries) plus a list of common contaminants and all the corresponding decoy entries [2]. For peptide identification a precursor ion mass tolerance of 7 ppm was used for MS1 level, trypsin was chosen as enzyme and up to three missed cleavages were allowed. The fragment ion mass tolerance was set to 0.5 Da for MS2 spectra. Oxidation of methionine and N-terminal protein acetylation were used as variable modifications whereas carbamidomethylation on cysteines was set as a fixed modification. False discovery rate (FDR) in peptide identification was set to a maximum of 5%. Finally, the raw proteomics data have been deposited to the ProteomeXchange Consortium via the PRIDE partner repository with the dataset identifier PXD015619.

**Supplementary Figures**

**
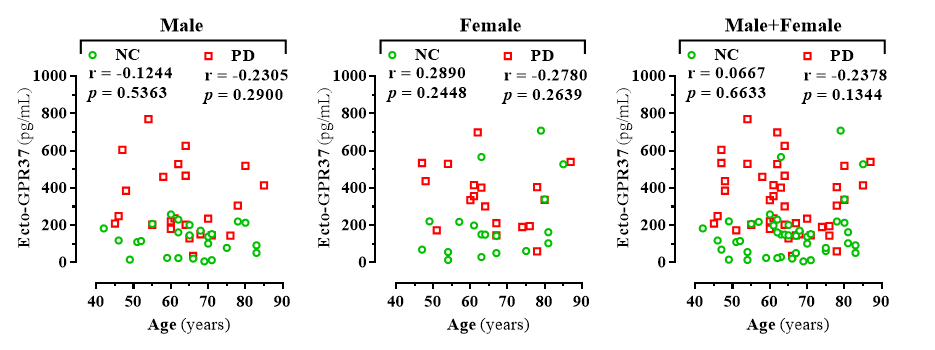
Supplementary Fig. S1.**

**
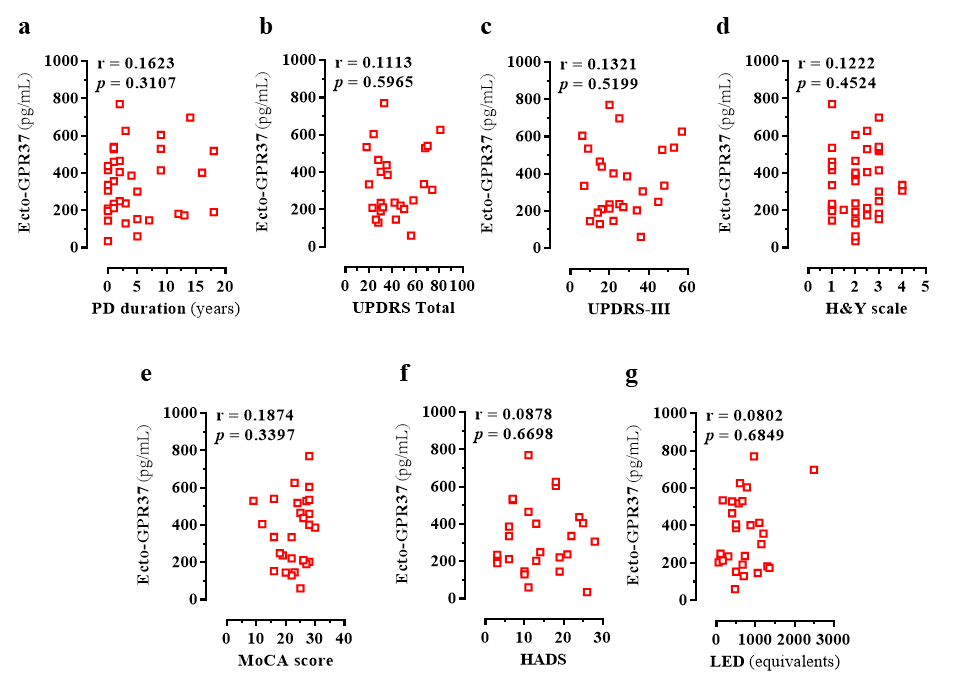
Supplementary Fig. S2.**

**References**

1. Chiva C, Olivella R, Borràs E, Espadas G, Pastor O, Solé A, et al. QCloud: A cloud-based quality control system for mass spectrometry-based proteomics laboratories. Lisacek F, editor. PloS one. 2018;13(1):e0189209.

2. Perkins DN, Pappin DJ, Creasy DM, Cottrell JS. Probability-based protein identification by searching sequence databases using mass spectrometry data. Electrophoresis. 1999;20(18):3551–67.
